# The Triage Role of Transabdominal Ultrasonography (TAUS) in the Diagnostic Management of Pancreatic and Distal Biliary Pathologies: A Comparative Efficacy Analysis with Endoscopic Ultrasonography (EUS)

**DOI:** 10.3390/diagnostics15232955

**Published:** 2025-11-21

**Authors:** Serkan Yaraş, Osman Özdoğan, Orhan Sezgin

**Affiliations:** Department of Internal Medicine (Gastroenterology), Faculty of Medicine, Mersin University, 33343 Mersin, Turkey; osmanozdogan2000@gmail.com (O.Ö.); drorhansezgin@gmail.com (O.S.)

**Keywords:** transabdominal ultrasonography, endoscopic ultrasonography, triage, pancreatobiliary lesions, diagnostic accuracy, lesion size

## Abstract

**Background/Objectives**: The diagnostic management of obstructive pancreatobiliary pathologies often leads to unnecessary invasive procedures and the overuse of costly imaging due to inherent diagnostic uncertainties. This dilemma highlights the need for a refined triaging strategy. This study aimed to compare the diagnostic competence and triage potential of Transabdominal Ultrasonography (TAUS)—a cost-effective, first-line method—with the efficacy of the invasive method, Endoscopic Ultrasonography (EUS). Our objective was to identify specific TAUS findings that could render EUS redundant or serve as a clinical guide for referral to EUS. **Methods**: This prospective study included patients evaluated for suspected pancreatobiliary lesions (December 2024–September 2025). Final diagnoses (gold standard) were established using pathology, tumor board decisions, other imaging, or ≥6 months clinical follow-up. TAUS was performed by one operator blinded to clinical data. EUS was immediately performed by a different operator, blinded to TAUS results and all other clinical data. Data were grouped into normal findings, solid masses, cystic lesions, chronic pancreatitis, distal cholangiocarcinoma/ampullary tumors, and choledocholithiasis. **Results**: A total of 204 patients were included. TAUS sensitivity (76.5%) was significantly lower than EUS (94.6%) (*p* < 0.001), but both showed high specificity (TAUS: 82.9%; EUS: 88.24%). TAUS performance varied greatly by lesion type: high for solid lesions (81.8%) and chronic pancreatitis (88.9%), but markedly lower for distal common bile duct lesions/ampullary tumors (57.1%; *p* = 0.006). In univariate analysis, BMI (*p* < 0.001), lesion size (*p* = 0.002), MPD dilation (*p* = 0.001), and localization (*p* < 0.001) were associated with TAUS success. Lesion size (OR = 1.049, *p* = 0.029) was the independent predictor in the multivariate analysis. TAUS detected common bile duct dilation in obstructive cases at a high rate (95.9%) but had statistically significantly lower success in reaching a definitive diagnosis (63.3%; *p* < 0.001). **Conclusions**: While TAUS lacks the overall sensitivity of EUS, its robust detection performance for solid lesions and chronic pancreatitis suggests that it can reduce the need for further investigation in selected cases. The TAUS detection success, associated with factors like BMI and lesion size, combined with its high rate of common bile duct dilation detection, establishes a reliable triage guideline for referring patients to advanced diagnostic procedures, primarily EUS, to confirm the definitive etiology.

## 1. Introduction

The pancreas is difficult to image due to its retroperitoneal location and heterogeneous pathologies. Diagnostic imaging therefore requires high sensitivity and specificity [1]. This is because pancreatic lesions include solid masses with malignant potential (such as pancreatic adenocarcinoma), various cystic neoplasms (serous, mucinous, IPMN), and inflammatory conditions (acute/chronic pancreatitis, pseudocysts), while the broader scope of pancreatobiliary disease includes choledocholithiasis (including microlithiasis) and periampullary tumors [2,3]. Despite technological advancements, the early detection and accurate staging of these lesions remain one of the main challenges in medical practice, as they directly impact patient prognosis [4,5].

Transabdominal Ultrasonography (TAUS) and Endoscopic Ultrasonography (EUS) are fundamental diagnostic tools for the evaluation of pancreatic and biliary tract diseases. TAUS is a non-invasive, widely accessible, cost-effective, and radiation-free imaging modality, and is generally accepted as the first-line screening tool in cases of clinical suspicion [6,7]. TAUS provides essential data for the initial detection of cystic and solid lesions, the presence of pseudocysts, and the assessment of major ductal changes associated with chronic pancreatitis [7,8,9,10,11]. In fact, TAUS has been shown to have a comparable level of efficacy to CT in the diagnosis and staging of chronic pancreatitis [12]. Furthermore, TAUS is a beneficial tool for the detection of biliary tract pathologies such as microlithiasis [13].

However, the efficacy of TAUS is frequently limited by patient anatomy, particularly due to obesity and bowel gas, which can lead to small or iso-echoic lesions, or tiny stones in the bile ducts, being easily overlooked [14,15,16]. These inherent limitations have caused EUS to assume a critical role in the diagnostic algorithm. Thanks to its close proximity to the pancreas, EUS has the ability to obtain images with millimeter resolution, thus offering higher sensitivity for small lesions compared to TAUS, CT, and MRI [17,18]. This superiority is due to its higher operating frequency and close proximity to the target organs within the gastrointestinal lumen, providing a substantially superior spatial resolution compared to TAUS. Specifically, in the diagnosis of choledocholithiasis, EUS provides a faster route to a definitive diagnosis by demonstrating a higher sensitivity than non-invasive methods like MRCP in some studies [19]. Most importantly, despite being an invasive procedure, EUS offers a fundamental advantage that other modalities cannot: the ability to achieve a definitive histopathological/cytological diagnosis for solid masses, biliary tract polyps, and cystic lesions through simultaneous EUS-guided fine-needle aspiration (EUS-FNA) or biopsy (EUS-FNB) [20]. EUS also shows superiority over TAUS, and even CT/MRI, in assessing morphological details such as nodules, wall thickening, and septations, which determine the malignant potential of cystic lesions [21,22].

Considering the varying efficacies and levels of invasiveness of these diagnostic methods, it is crucial to define the role of a high-quality TAUS within the diagnostic process and to determine when TAUS alone is sufficient to conclude the diagnostic process. Identifying the optimal utilization of non-invasive TAUS as a screening and triage tool compared to other advanced imaging modalities, including EUS, is a critical step for resource optimization and patient risk management [23,24]. Recent international guidelines, such as those from the European Society of Gastrointestinal Endoscopy (ESGE) on endoscopic management of common bile duct stones and endoscopic tissue sampling, underscore the need for efficient diagnostic algorithms but offer limited specific guidance on the integrated, full-spectrum triage role of TAUS versus EUS across the entire spectrum of obstructive pathologies [25,26]. This study aims to compare the diagnostic performances, advantages, and limitations of TAUS and EUS in the evaluation of pancreatic and biliary duct lesions, specifically in situations where TAUS is insufficient, and examines the relationship between TAUS findings and the need for EUS.

## 2. Materials and Methods

### 2.1. Study Design, Patient Population, and Ethical Approval

This study was conducted through the prospective analysis of data from patients evaluated at the Division of Gastroenterology at Mersin University Faculty of Medicine Hospital between December 2024 and September 2025 (Patient enrollment: December 2024 to March 2025 (3 months). Minimum follow-up: A minimum 6-month follow-up was required for all patients, which led to the study being definitively concluded and analyzed in September 2025).

The inclusion criteria for the study comprised patients aged 18 years and older who were evaluated for indications such as obstructive jaundice, unexplained upper abdominal pain, or suspicion of a pancreatobiliary mass/lesion. Written informed consent was obtained from all included patients, confirming their agreement to participate in the study and adhere to the specified diagnostic follow-up protocol (including final diagnosis verification via surgery, biopsy, or clinical follow-up). The complete availability of study variables such as BMI, lesion size, localization, and all laboratory parameters (CEA, CA 19-9, liver function tests, etc.) either prior to or concurrent with the EUS examination was also required for inclusion.

The exclusion criteria encompassed factors such as prior pancreatobiliary surgical resection or the presence of a metallic biliary stent, which could impair the quality of diagnostic imaging. Furthermore, patients with acute inflammatory processes, such as acute necrotizing pancreatitis, which would severely affect EUS and TAUS evaluations, or those with severe comorbidities constituting a contraindication to general anesthesia or endoscopic procedures (e.g., uncontrolled coagulopathy, advanced cardiac/respiratory failure), were also excluded. Finally, cases with missing demographic or laboratory variables (BMI, tumor markers, etc.) or those where the patient was anticipated to be unable to comply with long-term follow-up appointments were also not included in the study.

The research design was developed in accordance with the ethical guidelines of the Declaration of Helsinki and subsequently approved by the Mersin University Ethics Committee (Protocol No. 2024/1284; Approval Date: 25 December 2024).

### 2.2. Collection of Demographic and Laboratory Data

The demographic data (age and sex) and clinical risk factors (smoking status, alcohol consumption, and history of diabetes mellitus (DM)) of all patients included in the study were recorded prospectively at the study’s inception. Smoking status was categorized as a binary variable (smoker/non-smoker) based on the patients’ medical history. The non-smoker group included patients who had never smoked in their lifetime or whose cumulative smoking history was less than 10 pack-years. Patients with a smoking history exceeding this threshold (≥10 pack-years) were categorized as smokers, irrespective of their current status (current or former smoker). Alcohol consumption status was recorded as a binary variable (present/absent) based on patient self-report. The non-drinker category encompassed patients who had never consumed alcohol in their lifetime or who consumed alcohol less than once per week. An additional criterion for this category was the absence of a history of binge drinking (consumption of more than 5 standard drinks for men, or 4 for women, on a single occasion). Patients with regular or excessive alcohol consumption falling outside these criteria were included in the drinker category. Body mass index (BMI) was calculated in kg/m^2^ based on patients’ height and weight measurements.

All biochemical parameters (total bilirubin, GGT, ALP, ALT, AST) and tumor markers (CEA and CA 19-9) that were obtained within the last 48 h prior to imaging and met the study inclusion criteria were standardized and electronically recorded via the hospital automation system. Anamnestic data, such as a history of acute pancreatitis, were obtained through patient interviews and detailed review of existing medical records. All these data were then entered into specially designed case report forms (CRF) for the study and prepared for analysis. All collected data (demographic, clinical, laboratory, and imaging outcomes) were securely managed in a password-protected database (SPSS (statistical package for the social sciences) version 25.0 (IBM Corp., Armonk, NY, USA)) on the institutional server of Mersin University Faculty of Medicine Research Hospital. Data accuracy was rigorously ensured by double data entry performed independently by two researchers (SY and OÖ), with subsequent reconciliation to identify and resolve any transcription errors, thereby minimizing data entry bias.

### 2.3. Imaging Modalities and Technical Details

#### 2.3.1. Transabdominal Ultrasonography (TAUS)

All TAUS examinations were performed after the patients had fasted for at least 8 h, in accordance with a standardized protocol. Imaging was conducted using a Toshiba Applio-500^®^ (Toshiba Medical Systems Corporation, Tokyo, Japan) ultrasound system by a single gastroenterologist (OS) with over 10 years of TAUS experience. The performing operator was kept blinded to the patient’s clinical status and laboratory findings to minimize intra-observer variability. The TAUS assessment was carried out systematically to determine the etiology of the obstruction, measure bile duct/pancreatic duct dilation, and characterize the morphology of the pancreatic parenchyma and focal lesions.

#### 2.3.2. Endoscopic Ultrasonography (EUS)

All EUS procedures were performed immediately after the completion of the TAUS examination and on the same day, under standard patient sedation protocols. EUS was conducted by a second interventional gastroenterologist (SY), who was different from the TAUS operator (OS) and also had over 10 years of experience in the field. In our center, TAUS is performed by a gastroenterologist as a standard institutional practice, reflecting the integrated diagnostic workflow between endoscopy and ultrasonography. All examinations were conducted using a Toshiba Aplio-500^®^ system with a 3.5–5.0 MHz convex probe, with harmonic imaging and color Doppler employed when appropriate. Images were digitally stored for review and quality control. EUS examinations were performed using a linear-array Pentax EG-3870UTK^®^ (HOYA Corporation, Tokyo, Japan) scope in all cases. All EUS procedures were performed under sedoanalgesia using midazolam and pethidine. To prevent observer bias and information leakage during the procedure, a third investigator (OÖ) was assigned as a coordinator. OÖ ensured that the EUS operator (SY) remained completely blinded to the patient’s clinical status, laboratory findings, and the reports of other prior radiological images, including TAUS. This arrangement ensured the methodological validity of the study. The EUS assessment systematically focused on local and regional staging of the lesion, determination of the obstruction etiology, measurement of bile duct/pancreatic duct dilation, examination of the lesion’s relationship with the gastrointestinal wall, and characterization of paralesional vascular structures (e.g., portal and splenic veins) for signs of invasion.

### 2.4. Diagnostic Category and Subgroup Classification

To enhance the analytical scope of our study, the patients’ final diagnoses, confirmed by surgical pathology or clinical follow-up, were classified into five main groups from the collected 12 sub-diagnostic categories to represent the pancreatobiliary pathology spectrum. These groups are as follows:

Normal (Group 0): Comprised control patients in whom no pathology was detected during imaging or further evaluation.

Solid Lesions (Group 1): Consisted of the sub-diagnoses of adenocarcinoma (Adeno Ca), neuroendocrine tumor (NET), and benign solid lesion. The benign solid lesion subgroup included non-neoplastic entities such as focal inflammatory masses related to chronic pancreatitis, autoimmune pancreatitis, and focal fat-sparing areas confirmed as benign on follow-up or histopathology.

Cystic lesions (Group 2): Included cystic pancreatobiliary lesions, encompassing simple cyst, neoplastic cyst (such as intraductal papillary mucinous neoplasm (IPMN)), and pseudocyst.

Chronic pancreatitis (Group 3): Consisted of the single sub-diagnosis of chronic pancreatitis.

Malignant distal biliary obstructions (Group 4): Grouped from the sub-diagnoses of ampullary tumor and cholangiocarcinoma.

Lithiasis category (Group 5): Choledocholithiasis and microlithiasis diagnoses, which hold a significant place in the etiology of obstructive jaundice, were evaluated separately as subcategories under the lithiasis category for this analysis.

### 2.5. Gold Standard and Final Diagnosis (Definitive Diagnostic Methods)

The patients’ definitive diagnoses (gold standard) were established by at least one of the following methods in order to avoid reliance on a single modality and ensure diagnostic accuracy. These methods were prioritized according to their level of certainty:

Histopathological confirmation (highest priority): Results obtained from the histopathological and/or cytological examination of surgical resection specimens or fine-needle aspiration/biopsy (EUS-FNA/FNB) material acquired under EUS guidance.

Endoscopic retrograde cholangiopancreatography (ERCP) and lithiasis confirmation: Definitive diagnostic findings obtained via ERCP or the successful outcome of the therapeutic stone extraction procedure for diagnoses of obstructive jaundice etiology or biliary lithiasis.

Multidisciplinary board decision: tumor board (oncology board) decisions, involving radiology, gastroenterology, and oncology departments, based on advanced imaging findings, particularly for inoperable malignant lesions where histopathological evidence was deemed clinically unsuitable.

Clinical and radiological follow-up: Results of clinical and radiological progression (or stability) over a minimum period of 6 months, in conjunction with magnetic resonance cholangiopancreatography (MR/MRCP) and/or computed tomography (CT) findings, for cases of radiologically benign lesions and chronic pancreatitis. The follow-up protocol included clinical assessment every 3 months (detailed history, physical examination, and monitoring of specific biochemical markers such as CA 19-9, liver function tests, and C-reactive protein) and Transabdominal Ultrasonography (TAUS) at the 3rd and 6th month. CT or MRI was reserved if there was clinical or biochemical progression or if TAUS findings were equivocal.

This entire set of criteria ensured the reliability and validity of the final outcomes used for comparing diagnostic success in our study.

### 2.6. Statistical Analysis

All statistical analyses were performed using SPSS (statistical package for the social sciences) version 25.0 (IBM Corp., Armonk, NY, USA). At the beginning of the study, the entire cohort was divided into two main groups—patients with pancreatobiliary lesions and patients with normal/no lesions—and compared in terms of demographic (age, sex, BMI) and laboratory findings (ALT, GGT, CA 19-9, etc.). The independent samples *T*-test or Mann–Whitney U test was applied for continuous variables depending on data distribution, while the Chi-Square test or Fisher’s exact test was used for categorical variables.

The diagnostic performances of TAUS and EUS were then compared. The sensitivity, specificity, positive predictive value (PPV), and negative predictive value (NPV) for both modalities were calculated with 95% confidence intervals (CIs), and the McNemar test was employed to assess the statistical difference between the proportions.

Finally, the primary objective of the study—the triage (screening/referral) role of TAUS in the diagnostic process—was evaluated. For this purpose, subgroups where TAUS was successful and unsuccessful in detecting lesions were analyzed using the same fundamental comparison tests. Variables that demonstrated a significant difference were included in univariate and subsequent multivariate logistic regression analyses to determine their independent predictive ability for USG detection status. Detection thresholds for important continuous variables were determined using the ROC curve (AUC) and the Youden J statistic. A *p*-value of less than 0.05 was considered statistically significant in all analyses.

## 3. Results

### 3.1. Demographic and Clinical Characteristics of the Patients

A total of 204 patients were included in the study (Normal group: *n* = 38; Pancreatobiliary lesion group: *n* = 166). There were no statistically significant differences between the groups in terms of age (60.3 ± 15.6 vs. 63.7 ± 11.2 years, *p* = 0.187) or sex (female: 48.7% vs. 51.2%, *p* = 0.78). Likewise, body mass index (BMI), smoking/alcohol use, and history of diabetes mellitus (DM) did not differ significantly between the two groups (*p* > 0.05). However, the frequency of a history of acute pancreatitis was higher in the pancreatobiliary lesion group (22.7% vs. 5.3%, *p* = 0.01) (Table 1).

When laboratory parameters were compared, significant differences were observed between the groups. All markers of cholestasis and hepatic injury (total bilirubin, ALP, GGT, ALT, AST) were higher in the lesion group compared to the normal group (*p* ≤ 0.002). Among tumor markers, both CEA (*p* = 0.04) and, notably, CA 19-9 (*p* < 0.001) levels were found to be significantly elevated in the pancreatobiliary lesion group (Table 1).

### 3.2. Comparison of the Diagnostic Performance of EUS and TAUS in Pancreatobiliary Lesions

**Figure 1:**
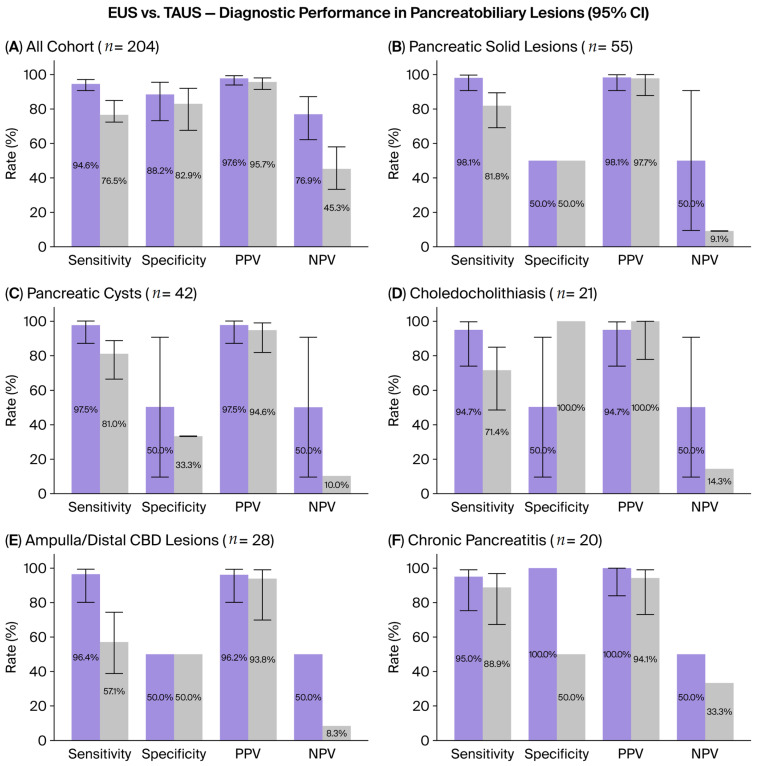
Diagnostic performance of EUS and TAUS in pancreatobiliary lesions (overall analysis and subgroup comparisons) (purple bars represents EUS, gray bars represents TAUS). Abbreviations: EUS, endoscopic ultrasonography; NPV, negative predictive value; PPV, positive predictive value; TAUS, Transabdominal Ultrasonography.

The diagnostic performance of EUS and TAUS was compared for the detection of all pancreatobiliary lesions. Among a total of 166 patients with confirmed lesions, TAUS successfully identified 127 cases but failed to detect 39. In contrast, EUS detected 157 lesions and missed only 9. Based on these findings, the diagnostic sensitivity of EUS was calculated as 94.6%, whereas that of TAUS was 76.5%. The corresponding specificity values were 88.2% for EUS and 82.9% for TAUS. The positive predictive value (PPV) was 97.6% for EUS and 95.7% for TAUS. Regarding the negative predictive value (NPV), EUS yielded 76.9%, whereas TAUS showed 45.3%. Overall, these results indicate that EUS demonstrates a higher sensitivity and NPV compared to TAUS (*p* < 0.001) (Table 2, Figure 1A). Specificity and Negative Predictive Value (NPV) could not be reliably calculated for several pathology subgroups due to the critically low number of true-negative cases within the enrolled patient cohort, which reflects the high pre-test probability inherent in the referral population of this study.

### 3.3. Comparison of TAUS and EUS in Subgroups of Pancreatobiliary Lesions

#### 3.3.1. Pancreatic Solid Lesions

Among the 55 pancreatic solid lesions, 45 were adenocarcinomas, 7 were neuroendocrine tumors, and the remaining 3 were benign lesions. In the evaluation of these solid pancreatic lesions, EUS demonstrated a significantly superior diagnostic performance compared with TAUS (*p* = 0.021). EUS achieved a sensitivity of 98.1%, surpassing the 81.8% sensitivity of TAUS. Regarding specificity, which is critical for diagnostic accuracy, both imaging modalities showed identical values (50%). In addition, the NPV of EUS (50%) was higher than that of TAUS (9.1%). The PPVs of both methods were high (EUS: 98.1%, TAUS: 97.7%) (Table 2, Figure 1B).

#### 3.3.2. Pancreatic Cystic Lesions

Among the 42 pancreatic cystic lesions, 14 were simple cysts, 25 were neoplastic cysts (the majority being IPMNs), and 3 were pseudocysts. In this subgroup, EUS demonstrated higher specificity compared with TAUS (97.5% vs. 80.9%). The sensitivity of EUS (97.8%) was also higher than that of TAUS (80.9%). The specificity values were 50% for EUS and 33.3% for TAUS. The PPV was 97.5% for EUS and 94.6% for TAUS. Furthermore, the NPV of EUS (50%) was calculated to be five times higher than that of TAUS (10%). Overall, EUS maintained a statistically significant superiority over TAUS in terms of sensitivity, specificity, PPV, and NPV in the evaluation of cystic pancreatic lesions (*p* = 0.012) (Table 2, Figure 1C).

#### 3.3.3. Choledocholithiasis

In a total of 21 patients with choledocholithiasis, EUS demonstrated a sensitivity of 94.7% and a specificity of 50.0%. TAUS showed a sensitivity of 71.4%. Due to the limited number of negative cases, the specificity (100.0%) and negative predictive value (NPV, 14.3%) for TAUS could not be reliably calculated (*). The difference in sensitivity between EUS and TAUS was statistically significant (*p* = 0.031). TAUS exhibited a slightly higher positive predictive value (PPV) (100.0%) than EUS (94.7%). The NPV of EUS was 50.0% (Table 2, Figure 1D).

#### 3.3.4. Ampullary/Distal Common Bile Duct Lesions

Among the ampullary/distal common bile duct lesions, 11 were ampullary tumors and 17 were distal cholangiocarcinomas. In this subgroup, the diagnostic performances of EUS and TAUS were compared. The sensitivity of EUS (96.4%) was nearly twice that of TAUS (57.1%), indicating a markedly superior performance. Both modalities demonstrated the same specificity (50.0%) and comparable PPVs (EUS: 96.2%, TAUS: 93.8%). However, the NPV of EUS (50.0%) was found to be higher and more reliable than that of TAUS (8.3%) (*p* = 0.006) (Table 2, Figure 1E).

#### 3.3.5. Comparison of Diagnostic Performance in Chronic Pancreatitis (CP)

In the diagnosis of chronic pancreatitis, the diagnostic sensitivity of EUS was calculated as 95%, whereas that of TAUS was 88.9%. The specificity was 100% for EUS and 50% for TAUS. The PPV was 100% for EUS and 94.1% for TAUS. The NPV of EUS was 50.0%, while for TAUS it was 33.3%. When these subgroup data were evaluated collectively, although EUS demonstrated higher diagnostic performance, the difference between the two modalities was not statistically significant (*p* = 0.500) (Table 2, Figure 1F).

### 3.4. The Triage Role of TAUS in Pancreatobiliary Lesions

Among the subgroups of pancreatobiliary lesions, the sensitivity of TAUS showed considerable variation. The highest sensitivity was observed in chronic pancreatitis (CP) at 88.9%, whereas the lowest sensitivity was found in ampullary/distal common bile duct (CBD) lesions at 57.1%. For the other subgroups, the sensitivities of TAUS were 81.8% in pancreatic solid lesions, 80.9% in pancreatic cysts, and 71.4% in choledocholithiasis. The specificity of TAUS could be calculated only for the overall cohort analysis, with a value of 82.9% (Table 2).

In most subgroups (pancreatic solid lesions, pancreatic cysts, choledocholithiasis, ampullary/distal CBD lesions, and chronic pancreatitis), specificity values could not be calculated due to an insufficient number of negative cases (Table 2).

TAUS demonstrated a high positive predictive capability, with PPVs of 100.0% for choledocholithiasis, 97.7% for pancreatic solid lesions, and 95.7% for the overall cohort. The lowest PPV was observed in ampullary/distal CBD lesions, at 93.8% (Table 2).

The NPV of TAUS was found to be low across all subgroups, with the lowest value observed in pancreatic cysts (10.0%) and the highest in chronic pancreatitis (33.3%). NPVs could not be calculated for choledocholithiasis and ampullary/distal CBD lesions due to an insufficient number of cases (Table 2).

To further assess the triage role of TAUS, demographic and anthropometric variables, laboratory parameters, and lesion-related factors such as size and localization were first compared using univariate statistical analyses (Table 3). As a result of this analysis, statistically significant associations were identified between TAUS performance and lesion size, lesion localization, and BMI. These significant parameters are presented in detail below under the respective subgroup headings.

#### 3.4.1. Lesion Size and the Detection Probability of TAUS in Pancreatic Solid and Cystic Lesions

Among the 97 pancreatic solid lesions, 81.44% (*n* = 79) were detected by TAUS, while 18.56% (*n* = 18) were not. The mean size of the detected lesions was 30.3 mm (range: 2.6–86 mm), whereas the mean size of undetected lesions was 17.6 mm (range: 1.5–64 mm), representing a statistically significant difference (*p* = 0.002) (Table 3). When analyzed by size category, the detection rate was found to increase proportionally with lesion size; the detection rate was 50% for lesions smaller than 10 mm, rising to 84% for those larger than 20 mm (Table 4).

**Figure 2:**
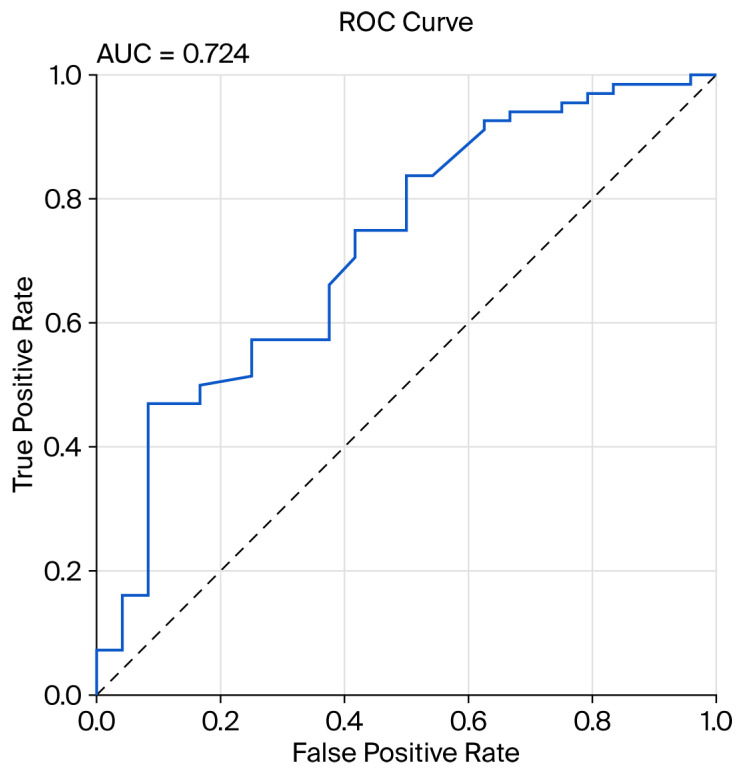
ROC curve illustrating the detectability of lesions by TAUS and the discriminative ability of the model (AUC).

The discriminative performance of the model was evaluated using ROC curve analysis, yielding an AUC value of 0.724 (Figure 2). The optimal cutoff point for lesion size corresponding to the highest detectability was determined to be 0.827, at which the model demonstrated a specificity of 91.7% and a sensitivity of 47.1%. According to the univariate logistic regression analysis, lesion size was identified as a statistically significant predictor of detectability by TAUS (*p* = 0.030). For each 1 mm increase in lesion size, the likelihood of detection by TAUS increased by approximately 4.9% (OR: 1.049, 95% CI: 1.005–1.096).

#### 3.4.2. Relationship Between Lesion Localization and the Detection Probability of TAUS in Pancreatic Solid and Cystic Lesions

In our dataset of cystic or solid pancreatic lesions in 97 patients, 57 were located in the head, 19 in the body, and 21 in the tail of the pancreas. The detection rate of these lesions by TAUS varied significantly depending on their localization (*p* < 0.001). Although the overall detection rate was high (81.4%), post hoc analyses revealed that this significant difference was primarily attributable to lesions located in the pancreatic tail. TAUS demonstrated high detection performance for lesions in the head (91.2%) and body (89.5%) of the pancreas, with no significant difference between these two regions (*p* = 0.811). However, the detection rate for tail lesions was markedly lower (47.6%). The probability of detecting a lesion in the pancreatic tail was statistically and significantly lower than that for lesions in the head and body regions (both *p* < 0.001) (Table 5).

#### 3.4.3. Relationship Between BMI and the Detection Probability of TAUS in Pancreatobiliary Lesions

**Figure 3:**
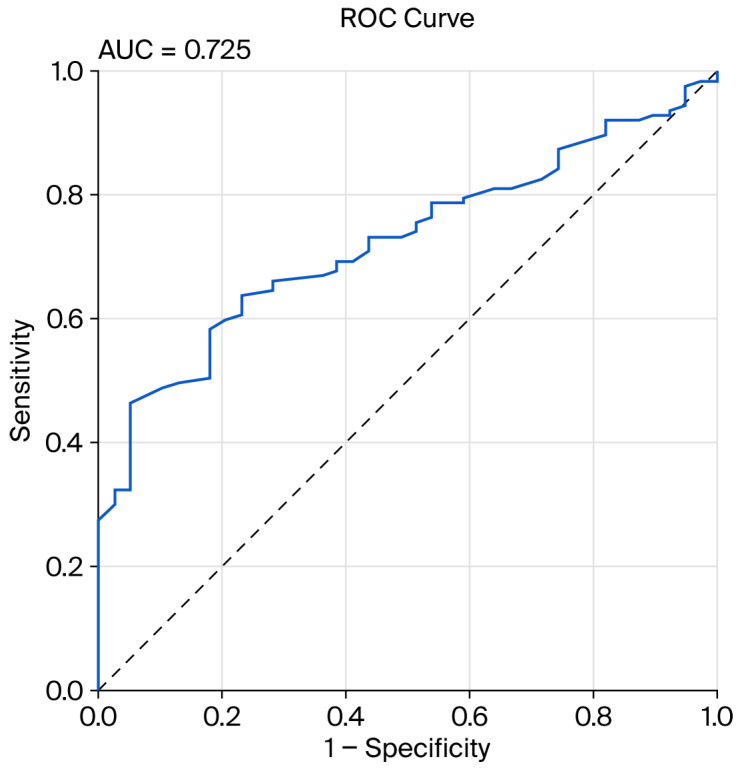
Receiver Operating Characteristic (ROC) curve demonstrating the diagnostic performance of body mass index (BMI) in predicting the detectability of pancreatic lesions by transabdominal ultrasonography (TAUS). The area under the curve (AUC) was 0.725, indicating good discriminative ability.

Among the 166 patients with pancreatobiliary lesions, those in whom TAUS failed to detect the lesion (*n* = 39) had a higher mean BMI (29.9 ± 3.2 kg/m^2^) compared to patients in whom the lesion was successfully detected (*n* = 127, 26.5 ± 4.7 kg/m^2^) (*p* < 0.001). To evaluate the relationship between BMI and the detection performance of TAUS, ROC curve analysis was performed. The analysis revealed that BMI had a significant predictive accuracy for lesion detectability by TAUS (AUC: 0.725, 95% CI: 0.647–0.803, *p* < 0.001). According to the Youden index, the optimal cutoff value was 28.4 kg/m^2^. In patients with BMI values above this threshold, the likelihood of lesion detection by TAUS was significantly reduced (sensitivity: 68%, specificity: 62%). The ROC curve demonstrates that, as body mass index increases, the diagnostic performance of transabdominal ultrasonography progressively declines (Figure 3).

#### 3.4.4. Relationship Between Main Pancreatic Duct and Common Bile Duct Dilatation and the Triaging Role of TAUS

Among the 166 patients with pancreatobiliary lesions included in the study, main pancreatic duct (MPD) dilatation was observed in 49 patients. The frequency of MPD dilatation was higher in the TAUS-successful group (45/127) compared with the TAUS-unsuccessful group (4/39) (*p* = 0.001).

Similarly, common bile duct (CBD) dilatation was detected in 66 of 166 patients (39.75%). However, there was no statistically significant difference in the frequency of CBD dilatation between the TAUS-successful group (48/127) and the TAUS-unsuccessful group (18/39) (*p* = 0.230) (Table 3). A subgroup analysis was performed for cases with CBD dilatation. Among the 49 patients diagnosed with choledocholithiasis or distal cholangiocarcinoma/ampullary tumor, all cases (100%) demonstrated CBD dilatation. TAUS successfully identified CBD dilatation in 95.9% (47/49) of these cases; however, it could establish the definitive diagnosis in only 63.3% (31/49) of them. Although TAUS could accurately detect CBD dilatation in nearly all evaluated cases (95.9%), its ability to translate this finding into a definitive diagnosis was significantly lower (63.3%, *p* < 0.001) (Table 3).

#### 3.4.5. Multivariate Logistic Regression Analysis of Independent Predictors of TAUS Success

To identify the independent predictors of TAUS success, a multivariate logistic regression analysis was performed using variables that were statistically significant (or clinically relevant) in the univariate analysis: BMI, lesion size, localization, and MPD dilatation. The results of this analysis are presented in Table 6.

Among the variables included in the model, only lesion size showed a statistically significant and independent association with TAUS success (*p* = 0.029). According to the analysis, each 1 mm increase in lesion size increased the likelihood of successful TAUS detection by approximately 4.9% (OR: 1.049; 95% CI: 1.005–1.096). This finding may reflect that larger lesions are more easily visualized by TAUS or that the relationship is influenced by interactions with other variables in the model.

Other variables—BMI (*p* = 0.234), localization (*p* = 0.849), and MPD dilatation (*p* = 0.116)—did not show a statistically significant independent effect on TAUS success (Table 6).

## 4. Discussion

Despite the development and use of various imaging modalities in the diagnosis of pancreatic lesions and pathologies causing obstruction in the common bile duct, challenges still persist. Early and accurate diagnosis is of critical importance in these pathologies, which are associated with high morbidity and mortality. Therefore, the efficacy and strategic positioning of imaging modalities within the diagnostic process are paramount. The main challenges encountered in the evaluation of lesions in this anatomical region are the increase in unnecessary invasive procedures due to diagnostic uncertainty and the ongoing debate regarding the competitive versus complementary roles of different modalities (TAUS, EUS). In this study, we compared the diagnostic competence and triage potential of TAUS—a non-invasive, widely available, and cost-effective method accepted as a first-line approach—with the efficacy and reliability of EUS, which is an invasive method that overcomes anatomical barriers with high resolution. Another focus of our study was to investigate whether TAUS could be used as a sufficient triage tool for certain pathologies alone. Furthermore, we aimed to evaluate which specific TAUS findings make the diagnostic process more efficient by potentially reducing unnecessary EUS procedures. Through this comparison, we sought to provide clinical data regarding the optimal sequence of applying diagnostic methods. In clinical reality, TAUS and EUS should not be viewed as competing methods but as complementary steps within a continuum of pancreatobiliary imaging. TAUS provides a broad initial overview and triage capability, whereas EUS adds high-resolution detail and tissue characterization, particularly in suspected malignancies.

In the overall cohort of 204 cases evaluated (38 normal, 166 with lesions), the diagnostic performance of EUS was substantially superior to TAUS (Sensitivity: 94.6% vs. 76.5%; NPV: 76.9% vs. 45.3%; *p* < 0.001). While both modalities showed high specificity and PPV (EUS 88.2%/97.6% and TAUS 82.9%/95.7%), these general data indicate that EUS is superior in the detection of pancreatobiliary lesions, particularly in terms of the reliability of a negative result. Given the limited number of comparative studies that encompass the entire lesion cohort, we discuss our findings by dividing them into clinically decisive subgroups (solid and cystic lesions) to better analyze the clinically specific roles of the two methods and the triage role of TAUS.

### 4.1. Efficacy of TAUS and EUS in Solid Pancreatic Lesions

In the diagnostic evaluation of solid pancreatic lesions (including adenocarcinoma, neuroendocrine tumors, and benign masses), TAUS and EUS assume complementary roles, establishing a critical triage mechanism within the diagnostic algorithm [7,27]. A systematic review and meta-analysis comparing imaging modalities reported that TAUS has a high sensitivity of 88% and a remarkable specificity of 94% in the diagnosis of pancreatic ductal adenocarcinoma (PDAC) [28].

In this context, the non-invasive nature and high positive predictive value (PPV) of TAUS guide the transition directly to EUS-Guided Fine-Needle Aspiration (EUS-FNA) for definitive diagnosis and staging in cases where it detects obstructive malignancy with high confidence [7]. However, the results of our study indicate that the performance of TAUS remains significantly lower than that of EUS in the evaluation of solid pancreatic lesions (*p* = 0.021). While EUS demonstrated a remarkably high sensitivity value, the sensitivity of TAUS remained at a substantially lower level. Crucially, the NPV of EUS (50%) is markedly higher than that of TAUS (9.1%). This finding supports the notion that low confidence should be placed in a negative TAUS result, underscoring the indispensable role of EUS in ruling out a mass.

The efficacy of TAUS is severely limited in challenging pathologies such as isoattenuating pancreatic carcinomas, where TAUS could directly visualize the mass in only 21% of cases [14]. This necessitates the involvement of EUS as an indispensable step due to its superior spatial resolution [27]. Critically, although TAUS could detect secondary findings (such as bile duct dilation) in isoattenuating PC in 88% of cases, it was able to detect Main Pancreatic Duct (MPD) dilation—one of the most sensitive indicators of early and occult malignancy—in only 13% of cases. In contrast, EUS detected MPD dilation significantly more frequently (58%) in the same group of isoattenuating lesions [14]. These findings reinforce the low TAUS sensitivity and low NPV observed in our own study, demonstrating that transition to EUS is mandatory in all scenarios where TAUS is insufficient or where critical risk factors such as high clinical suspicion, marked biliary tract dilation, or MPD dilation are present, even if a clear mass is not detected by TAUS [7,9,14].

The superior resolution provided by EUS, despite its invasive nature, enables the detection of small lesions. Furthermore, in lesion characterization where conventional EUS may be insufficient, advanced techniques such as Contrast-Harmonic EUS (CH-EUS) increase diagnostic certainty in differentiating between PDAC (hypovascular) and neuroendocrine tumors (hypervascular) [27]. Consequently, while TAUS serves as an effective filter due to its high specificity, EUS remains an indispensable diagnostic standard, offering the definitive diagnostic power required to reveal occult masses (especially in the presence of indirect signs), perform local staging, and provide histological confirmation. Clinical triage suggestion for solid lesions:

1. If TAUS is sufficient: If a clear TAUS image detects an obstructive mass with high confidence (high PPV), the patient should be presented directly to the multidisciplinary board for definitive diagnosis and treatment planning, and then referred for advanced staging workup (CT/MR) and/or EUS-FNA/CH-EUS or surgery as required.
2. If TAUS is insufficient or shows indirect findings: When pancreatic imaging via TAUS is insufficient, or when a clear mass is not detected but critical secondary findings such as high clinical suspicion, significant bile duct dilation, or main pancreatic duct (MPD) dilation (≥2.5 mm) are present, a transition to cross-sectional radiological modalities and/or EUS/EUS-FNA should be recommended for definitive diagnosis and local staging to rule out occult and isoattenuating malignancies.

### 4.2. Evaluation of Pancreatic Cystic Lesions

The evaluation of pancreatic cystic lesions presents significant challenges in clinical practice due to their broad spectrum of malignant potential. In the literature, a systematic comparative study by Hentschel et al. (2022) found the correct identification rate of pancreatic cystic lesions by TAUS to be only 31%; specifically, they reported that its sensitivity for detecting IPMNs was limited to 54% and that inter-observer agreement was quite low (0.093) [29]. This outcome indicates that TAUS is insufficient, particularly in the assessment of small and complex cystic lesions. Byrne et al. (2002), on the other hand, stated that pancreatic pseudocysts develop in less than 5% of acute pancreatitis cases and 20–40% of chronic pancreatitis patients [30]. While most of these lesions can be detected by transabdominal ultrasound, CT, or MR, they emphasized that EUS significantly improves diagnostic performance thanks to its superiority in morphological differential diagnosis and its high accuracy in ruling out malignancy via EUS-guided fine-needle aspiration.

The findings of our study, while consistent with literature demonstrating the general difficulties of TAUS in evaluating cystic lesions, indicate that TAUS offers significant diagnostic potential depending on lesion localization. Our findings support the role of TAUS as an initial detector. However, the definitive characterization and risk stratification of pancreatic cystic lesions, as outlined in international guidelines like the revised Fukuoka or European evidence-based guidelines, almost invariably require the superior morphological assessment and cyst fluid acquisition capabilities of EUS [31,32]. In the cystic lesion subgroup (*n* = 42), although the sensitivity of TAUS was found to be at a satisfactory level, EUS maintained a significant superiority over TAUS in sensitivity and all other diagnostic parameters (*p* = 0.012). Supporting the triage efficacy of TAUS, high detection success was achieved with TAUS in the head (91.2%) and body (89.5%) regions of the pancreas (the difference between them was non-significant). However, the main limitation of this success is its marked decrease in scenarios where the lesion is located specifically in the tail region (detection rate of 47.6%) and in small lesions (detection rate of 50% for those <10 mm) (*p* < 0.001). Despite this, the most critical reason for the low confidence in a negative TAUS result is that its negative predictive value is only one-fifth of the NPV of EUS. In conclusion, although TAUS constitutes an important first step in detecting cystic lesions, particularly in the head and body regions, reliance on the superior performance of EUS is necessary to assess the clinical significance of these lesions and reliably rule out malignancy. Clinical triage suggestion for cystic lesions are as follows:

1. If TAUS shows positive findings: If a clear cystic lesion is visualized (especially in the pancreatic head/body), the patient should be referred for advanced characterization modalities (MRCP/CT) and definitive diagnostic methods (EUS-FNA) to differentiate the lesion’s benign/malignant potential.
2. If TAUS is negative or imaging is insufficient: When TAUS imaging is insufficient (e.g., pancreatic tail or small lesions <10 mm), or when no lesion is detected but clinical suspicion persists, EUS/EUS-FNA examination is recommended for definitive diagnosis and to reliably rule out malignancy.

### 4.3. Role of TAUS and EUS in the Diagnosis of Choledocholithiasis

In the diagnostic evaluation of choledocholithiasis, TAUS assumes a critical role as the initial imaging modality due to its non-invasive nature. One study demonstrated that TAUS possesses significant predictive value, exhibiting strong agreement (Cohen’s Kappa = 0.748, *p* < 0.001) and a high correlation in diagnostic accuracy (Pearson r = 0.856) when compared to ERCP, which is considered the gold standard for detecting biliary tract obstructive lesions [33]. This triage role is strongly supported by the findings of our study. TAUS successfully detected dilation in nearly all cases with common bile duct dilation. This finding suggests that TAUS may be sufficient as a triage tool prior to therapeutic intervention by detecting obstructive lesions with high reliability. Furthermore, in the choledocholithiasis subgroup, the Positive Predictive Value (PPV) of TAUS (100.0%) demonstrated a higher performance than the PPV of EUS (94.7%), highlighting the strong diagnostic power of direct detection of a stone by TAUS.

However, the efficacy of TAUS is limited, particularly in situations where gas artifacts complicate imaging, such as in microlithiasis and the distal common bile duct (CBD) [30]. In our study, the sensitivity of TAUS (71.4%) was found to be statistically significantly lower than that of EUS (94.7%) (*p* = 0.031). This indicates the inadequacy of TAUS in detecting the presence of stones and the necessity to transition to EUS when clinical suspicion is high. EUS is recognized as a highly effective invasive diagnostic tool in the detection of common bile duct stones. A recent comprehensive meta-analysis confirmed the high diagnostic accuracy of EUS for choledocholithiasis, reporting a pooled sensitivity of 0.96 and specificity of 0.90, making it a robust alternative to MRCP [34]. Another study revealed that EUS demonstrated a significant superiority over TAUS, especially in the detection of microlithiasis in the gallbladder and the common bile duct [35].

In light of these data, the optimization of the diagnostic algorithm becomes clear: TAUS is an indispensable first-line triage tool due to its non-invasive nature and its high reliability in detecting obstruction and large stones (high PPV). In cases where TAUS detects a clear obstructive lesion, transition can be made directly to therapeutic ERCP or definitive treatment planning, bypassing the need for diagnostic EUS. Conversely, in situations where TAUS is insufficient (low sensitivity) or where high clinical suspicion persists, such as in microlithiasis, EUS is the preferred invasive upgrade step, utilizing its high sensitivity and specificity to prevent unnecessary ERCP and establish a definitive diagnosis. Clinical triage suggestion for choledocholithiasis:

1. If TAUS shows positive findings: If a clear TAUS image detects significant common bile duct dilation and stone presence, the patient can be referred directly for therapeutic ERCP or definitive treatment planning, bypassing the need for diagnostic EUS.
2. If TAUS is negative or insufficient: In cases where imaging is insufficient (especially if microlithiasis or distal CBD is suspected), EUS and/or MRCP should be utilized to alleviate high clinical suspicion and establish a definitive diagnosis, considering the superior diagnostic power of these methods.

### 4.4. Malignant Distal Biliary Obstructions (Cholangiocarcinoma and Ampullary Tumor)

In the diagnostic evaluation of malignant distal biliary obstruction causes, such as distal cholangiocarcinoma and ampullary tumor, TAUS and EUS assume sequential and complementary roles [36,37]. TAUS has been reported to show strong agreement with ERCP (Pearson r = 0.856), which is the gold standard for detecting obstructive biliary lesions [29]. For an ampullary tumor/mass, this concordance was reported as 71.4%, suggesting that TAUS can detect these lesions with high confidence and guide the transition to further investigation [33].

Our study demonstrates the diagnostic superiority of EUS by showing that its sensitivity (96.4%) in ampullary/distal CBD lesions is nearly double that of TAUS (57.1%) (*p* = 0.006). Data in the literature also report the low sensitivity of TAUS in the detection of distal CBD tumors [38]. However, the key finding that renders the triage role of TAUS absolutely indispensable is its success in detecting the indirect sign caused by the lesion, rather than the lesion itself. Common bile duct dilation was detected in all of our malignant and choledocholithiasis cases (100%), and TAUS was able to demonstrate this dilation with overwhelming success (95.9%). This clearly proves that, even though the direct malignancy diagnostic competence of TAUS is limited, it acts as the most reliable and least expensive filter for capturing cases that raise suspicion of malignancy and require further investigation.

In our study, while TAUS and EUS had similarly high positive predictive values (93.8% and 96.2%, respectively), the negative predictive value (NPV) of EUS (50.0%) was found to be significantly more reliable than the NPV of TAUS (8.33%) (*p* = 0.006). This finding indicates that a negative TAUS result is unreliable in ruling out malignancy. Furthermore, it demonstrates that EUS is an absolute necessity, thanks to its superior spatial resolution, for definitively identifying occult lesions in all cases where TAUS inadequately detects the etiology of malignant obstruction or where common bile duct dilation with an undetermined cause is observed. Clinical triage suggestions for malignant distal biliary obstructions are as follows:

1. If TAUS shows positive findings: In cases where TAUS detects the obstructive lesion itself along with common bile duct dilation with high confidence (based on the high PPV of TAUS), the patient can be referred to the multidisciplinary board and/or advanced cross-sectional radiological modalities (CT/MR), or EUS can be employed for further staging and treatment planning.
2. If TAUS only detects dilation: In all cases where TAUS cannot definitively establish the etiology of malignancy but detects common bile duct dilation—since this dilation finding is the strongest clue for the need for further diagnosis—EUS/EUS-FNA and/or cross-sectional radiological modalities (CT/MR) are recommended for definitive diagnosis and to rule out malignancy.

### 4.5. Chronic Pancreatitis and Imaging Strategy

In the diagnosis of chronic pancreatitis, some guidelines and publications recommend TAUS as the first-line method for determining the patient’s long-term prognosis and treatment stratification [39,40]. Our study provided strong data supporting this general consensus. Although the sensitivity of EUS (95%) in the diagnosis of chronic pancreatitis was numerically higher than that of TAUS (88.9%), no statistically significant difference was found between the two methods (*p* = 0.500). This finding suggests that TAUS can exhibit performance comparable to EUS in cases of chronic pancreatitis showing advanced morphological changes such as significant ductal dilation, calcification, or pseudocysts. Indeed, Nordaas et al. (2021) also showed that the diagnostic accuracy of TAUS is comparable to that of computed tomography (CT) [12]. Furthermore, Jung et al. (2025) supported the diagnostic potential of TAUS by revealing that advanced TAUS techniques, such as transabdominal ultrasound shear-wave elastography (TA-SWE), show a high degree of concordance with EUS [41].

However, in our study, we found the specificity of EUS (100%) to be significantly higher than the specificity of TAUS (50%). We also found the negative predictive value (NPV) of TAUS (33.3%) to be lower than that of EUS (50.0%). These metrics indicate that TAUS is not as definitive as EUS in identifying early morphological changes and ruling out malignancy, and it carries a higher risk of false positive/negative results.

In conclusion, TAUS is an indispensable triage tool in the initial imaging of patients with suspected chronic pancreatitis and in the follow-up of significant anatomical changes, owing to its high sensitivity and cost-effectiveness. This allows for the optimization of the diagnostic algorithm by reserving invasive methods like EUS for situations where clinical suspicion remains high, where early morphological changes are being investigated, or where a definitive diagnosis is required. Clinical triage suggestions for chronic pancreatitis are as follows:

1. If TAUS shows positive findings: If a clear TAUS image detects significant signs of chronic pancreatitis (pseudocyst, marked ductal dilation, or calcification), cross-sectional radiological modalities (CT/MR) can be performed for diagnostic certainty and staging, if deemed necessary.
2. If TAUS is negative or indeterminate: In situations where clinical suspicion remains strong but TAUS fails to detect significant changes or its diagnostic specificity is insufficient, EUS and/or cross-sectional radiological modalities (CT/MR) are recommended to detect early morphological signs, rule out malignancy, and establish a definitive diagnosis.

It should be noted that stratifying diagnostic performance strictly by benign or malignant outcomes was not the primary purpose of this study. Because both TAUS and EUS were performed prior to definitive histopathologic confirmation, post hoc separation into benign and malignant categories could introduce bias and reduce methodological integrity. Therefore, the present analysis emphasizes the complementary and functional diagnostic contribution of TAUS and EUS within the clinical decision-making pathway rather than histologic subtyping.

### 4.6. The General Triage Role of TAUS

Our study evaluated the potential and limitations of TAUS as a triage tool in the management of pancreatic lesions, revealing that the morphological characteristics of the lesion and certain patient-related factors influence the success of TAUS.

We found that the diagnostic success of TAUS varied significantly based on the type and localization of the lesion. In our study, TAUS demonstrated its effectiveness as a filtering tool with high sensitivity in pronounced pathologies like chronic pancreatitis (88.9%) and pancreatic solid lesions (81.8%). However, sensitivity dropped to 57.1% in ampullary/distal Common Bile Duct (CBD) lesions (*p* = 0.006), indicating the inadequacy of TAUS in hard-to-reach areas. Similarly, the marked decrease in the detection rate in the pancreatic tail region (47.6%, *p* < 0.001) supports this observation. Furthermore, the diagnostic performance of TAUS was found to decrease with increasing Body Mass Index (BMI > 28.4 kg/m^2^), suggesting that advanced imaging (EUS/CT/MR) should be prioritized in obese patients (AUC: 0.725). However, the main finding that makes the triage role of TAUS absolutely indispensable is its success in detecting lesion size and the indirect signs it causes. As a result of our multivariate analysis, unlike other clinical variables that were significant in univariate analysis, only pathology size (OR: 1.049; 95% CI: 1.005–1.096; *p* = 0.029) was identified as a statistically significant and independent predictor of TAUS success. The mean size of detected lesions (30.3 mm) was significantly higher than that of non-detected lesions (17.6 mm) (*p* = 0.002), indicating that lesion size is the most critical morphological criterion for the triage potential of TAUS. This dependence on lesion size is consistent with the physical limitations of ultrasound, where resolution and beam penetration directly impact the ability to visualize smaller objects, particularly in a retroperitoneal organ like the pancreas. This finding is in strong concordance with the results of studies by Jeon et al. (2018) (OR: 1.070; 95% CI: 1.044–1.096; *p* < 0.001) and Choi et al. (2020) (OR: 1.070; *p* < 0.001), which reported that lesion size independently affects TAUS success [42,43]. Sahani et al. (2013) also stated that the role of TAUS in the evaluation of cystic lesions is dependent on lesion size [44]. Therefore, TAUS is an ideal tool for quickly filtering out the large lesions that require the most urgent surgical and oncological intervention, rather than the small lesions that are most at risk of being missed.

In addition, common bile duct dilation was successfully detected in almost all of our cases with obstructive pathology (95.9%). This clearly proves that while TAUS leaves the definitive etiological diagnosis (success rate of 63.3%) to advanced imaging, it serves as the most reliable and economical filter for detecting patients who raise suspicion of malignancy or severe obstruction and require further diagnosis. However, the consistently low negative predictive value (NPV: 10.0–33.3%) across all subgroups emphasizes that a negative result is unreliable in ruling out serious pathology and that the need for further investigation (EUS/CT/MR) absolutely continues when clinical suspicion persists, as also noted by Reddymasu et al. (2011) and Zhang et al. (2025) [45,46].

### 4.7. Study Limitations and Strengths

A significant limitation of our study is the issue of high prevalence encountered in the subgroup analyses. This, combined with the low patient numbers, led to a widening of the confidence intervals, particularly for parameters like NPV and specificity, resulting in statistically unstable outcomes. Consequently, these parameters weaken the scientific strength of the cross-modality comparison (EUS vs. TAUS). Furthermore, as this study was conducted in a tertiary gastroenterology referral center, the patient population inherently included individuals with a higher pre-test probability of pancreatobiliary disease. Therefore, the diagnostic performance values reported should be interpreted within this context. Additionally, while the use of a single, experienced operator for each modality minimized the risk of inter-observer variability (a key strength), it may limit the direct generalizability of the reported exact sensitivity and specificity values to centers with operators of widely varying experience levels. The strength of our study, however, lies in its inclusion of nearly all pancreatobiliary lesions and its status as one of the few studies in the literature that discusses the triage role of TAUS.

## 5. Conclusions

Although TAUS does not reach the level of EUS in terms of overall sensitivity, it remains an effective method, especially in the detection of prominent pathologies such as solid lesions and chronic pancreatitis, and may reduce the need for further invasive evaluation in some cases. Due to its non-invasive and cost-effective nature, it offers a suitable method for initial assessment, demonstrating high sensitivity and positive predictive value in the diagnosis of large and distinct lesions. While TAUS is effective in cases of chronic pancreatitis, solid or cystic lesions located in the head and body, and common bile duct dilation, negative results are unreliable for small, tail-located, or low-contrast lesions; in these scenarios, EUS or advanced imaging techniques are required. EUS, with its high resolution and capacity for histological confirmation, provides diagnostic certainty and should only be employed in situations where TAUS is insufficient or when additional evaluation is needed. This strategy enhances the efficiency of the diagnostic process, reduces unnecessary invasive procedures, and optimizes resource utilization.

## Figures and Tables

**Table 1:** Comparison of demographic, clinical, and laboratory data between the study groups.

Parameter	Normal Group (*n* = 38)	Group with Pancreaticobiliary Lesion (*n* = 166)	*p*-Value
Age (years)	60.3 ± 15.6	63.7 ± 11.2	0.187
Gender (female, %)	48.7	51.2	0.780
BMI (kg/m^2^)	27.5 ± 3.9	27.3 ± 4.1	0.732
Smoking (%)	22.3	28.7	0.330
Alcohol Consump. (%)	12.5	18.1	0.270
DM (%)	9.4	16.0	0.180
AP history (%)	5.3	22.7	0.010
Total Bil (mg/dL)	0.85 ± 0.35	2.69 ± 3.01	0.002
GGT (U/L)	66.1 ± 78.4	188.2 ± 160.5	<0.001
ALP (U/L)	97.9 ± 41.1	226.7 ± 103.5	<0.001
ALT (U/L)	31.4 ± 16.5	82.8 ± 64.1	<0.001
AST (U/L)	29.0 ± 14.9	71.5 ± 58.5	0.001
CEA (ng/mL)	1.7 ± 1.2	4.8 ± 11.2	0.040
CA 19-9 (U/mL)	23.5 ± 18.1	246.7 ± 400.2	<0.001

Abbreviations: ALP, Alkaline Phosphatase; ALT, Alanine Aminotransferase; AP, Acute Pancreatitis; AST, Aspartate Aminotransferase; BMI, Body Mass Index; CA 19-9, Carbohydrate Antigen 19-9; CEA, Carcinoembryonic Antigen; DM, Diabetes Mellitus; GGT, Gamma-Glutamyl Transferase; U/L, Units per Liter.

**Table 2:** Comparison of the diagnostic performance parameters (sensitivity, specificity, PPV, NPV) of EUS and TAUS in all pancreatobiliary lesions and specific pathological subgroups (*: Not calculable due to insufficient cases).

Group	Modality	Sensitivity (%) (95% CI)	Specificity (%) (95% CI)	PPV (%) (95% CI)	NPV (%) (95% CI)	*p*
All cohort (*n* = 204)	TAUS	76.5 (72.2–84.8)	82.9 (67.4–91.8)	95.7 (91.3–97.9)	45.3 (33.2–57.9)	0.000
	EUS	94.6 (90.6–97.0)	88.2 (73.2–95.3)	97.6 (93.8–99.1)	76.9 (62.1–87.1)	
Pancreatic solid lesion (*n* = 55)	TAUS	81.8 (69.1–89.3)	50.0 (*)	97.7 (87.9–99.9)	9.1 (*)	0.021
	EUS	98.1 (90.6–99.8)	50.0 (9.5–90.6)	98.1 (90.6–99.8)	50.0 (9.5–90.6)	
Pancreatic cyst (*n* = 42)	TAUS	80.9 (66.3–88.6)	33.3 (*)	94.6 (81.7–98.7)	10.0 (*)	0.012
	EUS	97.5 (86.8–99.9)	50.0 (9.5–90.6)	97.5 (86.8–99.9)	50.0 (9.5–90.6)	
Choledocholithiasis (*n* = 21)	TAUS	71.4 (48.5–84.8)	100.0 (*)	100.0 (77.6–100)	14.3 (*)	0.031
	EUS	94.7 (74.0–99.4)	50.0 (9.5–90.5)	94.7 (74.0–99.4)	50.0 (9.5–90.5)	
Ampulla/distal common bile duct (*n* = 28)	TAUS	57.1 (38.8–74.5)	50.0 (*)	93.8 (69.8–99.2)	8.3 (*)	0.006
	EUS	96.4 (79.9–99.3)	50.0 (*)	96.2 (79.9–99.3)	50.0 (*)	
Chronic pancreatitis (*n* = 20)	TAUS	88.9 (67.2–96.9)	50.0 (*)	94.1 (73.0–98.9)	33.3 (*)	0.500
	EUS	95.0 (75.4–99.2)	100.0 (*)	100.0 (83.9–100)	50.0 (*)	

* Not calculable due to insufficient cases. Abbreviations: EUS, endoscopic ultrasonography; NPV, negative predictive value; PPV, positive predictive value; TAUS, Transabdominal Ultrasonography.

**Table 3:** Univariate analysis of factors associated with Transabdominal Ultrasonography (TAUS) detection success (triage role).

Parameter	TAUS (Success)	TAUS (Failure)	*p*-Value
Age (years) (mean ± SD)	62.8 ± 14.7	66.6 ± 12.9	0.140
Sex (female, %)	51.2%	53.8%	0.769
BMI (kg/m^2^) (mean ± SD)	29.9 ± 3.2	26.5 ± 4.7	<0.001
Smoking (%)	29.9%	25.6%	0.599
Alcohol_C (%)	19.6%	12.8%	0.341
CEA (ng/mL) (mean ± SD)	5.2 ± 10.8	3.5 ± 11.9	0.403
CA 19-9 (U/mL) (mean ± SD)	259.8 ± 395	204.1 ± 425	0.448
ALT (U/L) (mean ± SD)	86.2 ± 61.8	73.3 ± 69.3	0.270
AST (U/L) (mean ± SD)	73.1 ± 55.9	62.6 ± 64.1	0.323
GGT (U/L) (mean ± SD)	195.6 ± 157.1	154.1 ± 166.7	0.157
ALP (U/L) (mean ± SD)	230.1 ± 99.8	215.6 ± 111.2	0.441
Size (mm) (mean ± SD)	30.3 ± 17.4	17.6 ± 14.1	0.002
Localization * (1/2)	90.8%/47.6%	9.2%/52.4%	<0.001
MPD frequency (%)	35.4%	10.3%	0.001
CD frequency (%)	37.7%	46.1%	0.230

* (1: head and body of pancreas, 2: tail of pancreas. For detailed localization evaluation, see Section 2.3.2). Abbreviations: Alcohol_C, Alcohol Consumption; ALP, Alkaline Phosphatase; ALT, Alanine Aminotransferase; AST, Aspartate Aminotransferase; BMI, Body Mass Index; CA 19-9, Carbohydrate Antigen 19-9; CD, Choledocal Dilation; CEA, Carcinoembryonic Antigen; GGT, Gamma-Glutamyl Transferase; MPD, Main Pancreatic Duct; SD, Standard Deviation; TAUS, Transabdominal Ultrasonography; U/L, Units per Liter.

**Table 4:** TAUS detection rates of pancreatic solid and cystic lesions according to maximum lesion diameter.

Lesion Size Category (mm)	Number of Cases (*n*)	TAUS Detection Rate
<10	22	0.5
10–20	20	0.75
>20	55	0.84

**Table 5:** Impact of lesion localization (head, body, tail) on the detection success of Transabdominal Ultrasonography (TAUS).

USG Status	Localization			
	Head *n* (%)	Body *n* (%)	Tail *n* (%)	*p*-Value
USG detected	52 (91.2%)	17 (89.5%)	10 (47.6%)	<0.001
USG failed	5 (8.8%)	2 (10.5%)	11 (52.4%)	
Total	57 (100%)	19 (100%)	21 (100%)	

**Table 6:** Multivariate logistic regression analysis of variables predicting the diagnostic success of TAUS.

Variable (Predictor)	Odds Ratio (Exp (B))	95% Confidence Interval (CI)	*p*-Value
Pathology Size (mm)	1.049	1.005–1.096	0.029
BMI (kg/m^2^)	1.106	0.937–1.307	0.234
Localization (1 vs. Ref.)	0.875	0.221–3.468	0.849
MPD Dilatation (Yes vs. No)	3.372	0.740–15.365	0.116

Abbreviations: BMI, body mass index; CI, confidence interval; Exp (B), exponential of B (odds ratio); MPD, main pancreatic duct; vs., versus.

## Data Availability

The datasets generated and analyzed for the current study are included within the article.

## Abbreviations

The following abbreviations are used in this manuscript:

BMI	Body mass index
CBD	Common bile duct
CT	Computed tomography
DM	Diabetes mellitus
ERCP	Endoscopic retrograde cholangiopancreatography
EUS	Endoscopic ultrasonography
IPMN	Intraductal papillary mucinous neoplasm
MPD	Main pancreatic duct
MRCP	Magnetic resonance cholangiopancreatography
MRI	Magnetic resonance imaging
NPV	Negative predictive value
PPV	Positive predictive value
TAUS	Transabdominal ultrasonography
